# Pleistocene origin and colonization history of *Lobelia columnaris* Hook. f. (Campanulaceae: Lobelioideae) across sky islands of West Central Africa

**DOI:** 10.1002/ece3.8256

**Published:** 2021-10-20

**Authors:** Miguel A. Pérez‐Pérez, Wen‐Bin Yu

**Affiliations:** ^1^ Department of Biological Sciences Northern Arizona University Flagstaff Arizona USA; ^2^ Department of Biodiversity, Earth & Environmental Science Drexel University Philadelphia Pennsylvania USA; ^3^ Center for Integrative Conservation Xishuangbanna Tropical Botanical Garden Mengla China; ^4^ Center of Conservation Biology, Core Botanical Gardens Chinese Academy of Sciences Mengla China; ^5^ Southeast Asia Biodiversity Research Institute Chinese Academy of Science Yezin Myanmar

**Keywords:** Bioko, Cameroon, Cameroon Volcanic Line, Campanulaceae: Lobelioideae, *Lobelia*, phylogenomics, plastomes, sky islands, West Central Africa

## Abstract

We aimed to infer ancestral area and historical colonization of *Lobelia columnaris* in the sky islands of Bioko and Cameroon through dated phylogeny using chloroplast genomes. Specifically, we aim to answer the following questions: (1) What are the phylogenetic relationships among Bioko Island and Cameroon populations? (2) Are the older populations found in the older sky islands? We assembled novel plastomes from 20 individuals of *L. columnaris* from 5 mountain systems. The plastome data were explored with phylogenetic analyses using Maximum Likelihood and Bayesian Inference. The populations of *L. columnaris* have a monophyletic origin, subdivided into three plastomes‐geographic clades. The plastid phylogenomic results and age of the sky islands indicate that *L. columnaris* colonized first along with the Cameroon Volcanic Line's young sky islands of Bioko. The crown group (1.54 Ma) split the population in Bioko and mainland Cameroon. It is possible that Bioko was the ancestral area and likely isolated during cold and dry conditions in forest refugia. Presumably, the colonization history occurred during the middle‐late Pleistocene from South Bioko's young sky island to North Bioko and the northern old sky islands in Cameroon. Furthermore, the central depression with lowland forest between North and South Bioko is a current geographic barrier that keeps separating the populations of Bioko from each other. Also, the shallow sea channel keeps isolated the populations of Bioko and the mainland populations. The Pleistocene climatic oscillations led to the divergence of the Cameroon and Bioko populations into three clades. *L. columnaris* colonized the older sky islands in mainland Cameroon after establishment in Bioko's younger sky islands. Contrary to expectations, the biogeography history was an inverse progression with respect to the age of the Afromontane sky islands.

## Graphical abstract

Lobelia columnaris colonized the sky island of West Africa during the Pleistocene. The colonization history of this giant lobelia was from the young sky islands through old sky islands in mainland Africa.

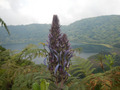

## 1 INTRODUCTION

Sky islands are continental habitats that are ecologically and environmentally isolated and surrounded by a “sea” of low‐elevation habitats that limit dispersal of lineages (Gupta et al., 2019). Sky islands are considered natural laboratories for studying evolutionary patterns and processes that lead to the accumulation of diversity (Cox et al., 2014). These isolated habitats are critical to investigate species colonization and in situ speciation in the Asteraceae, specifically the genus *Senecio* in sky islands of Eastern Africa (Kandziora et al., 2016). Moreover, Shola sky islands, Western Ghats, southern India are natural laboratories that offer the opportunity to study biogeographic gaps, elevational gradients, and host species barriers that drive the evolution of parasite community structure (Gupta et al., 2019). Indeed, heterogeneous landscapes from sky islands and sky island archipelagos are promoters of population genetic differentiation at spatial and temporal scales (Luo et al., 2016). Also, sky islands have operated like long‐term refugia and cradles of significant genetic structure caused by Pleistocene climatic changes (Mairal et al., 2017).

The Pleistocene's climate and environmental oscillations caused fragmentation and expansion of lowland tropical rain forests, mountain forests, and savannas of Upper Guinea and Lower Guinea, Africa (Duminil et al., 2015). These climate oscillations shaped Afromontane species distributions, such as *Lobelia giberroa*, which occurs on mountains with altitudes between 3500 and nearly 6000 m in East Africa (Kebede et al., 2007). Forest fragmentation during the Pleistocene was a driver of allopatric speciation in plants and animals. Likewise, forest refugia and rivers contributed to current diversity patterns in West Central Africa (Nicolas et al., 2012). Moreover, there was substantial volcanic activity in West Central African and East Africa during the Pliocene–Pleistocene epochs. For example, volcanic activity caused population expansion of endemic plants and vertebrates in the Taita Hills (Measey & Tolley, 2011).

**FIGURE 1:**
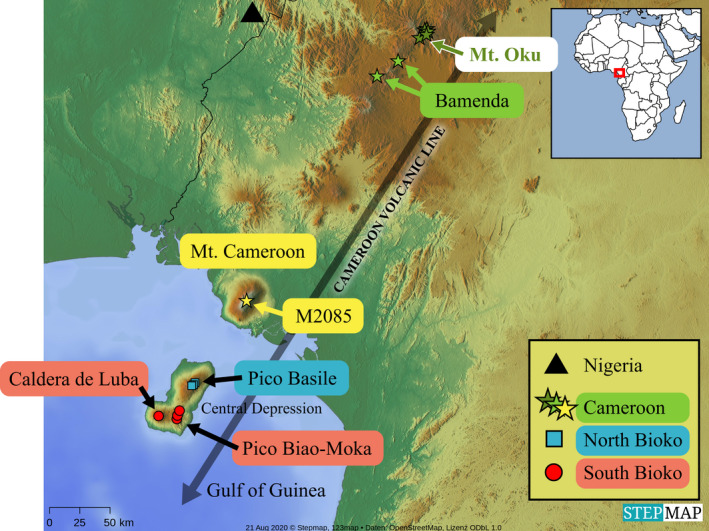
Geographic distribution of plastomes of *Lobelia columnaris*

The Cameroon Volcanic Line (CVL) is an 1800‐km SW‐NE topographical feature that extends from the Gulf of Guinea to the onshore central Cameroon (Adams et al., 2015). This volcanic chain was an ancient forest refuge in West Central Africa (Demenou et al., 2020). The CVL comprises plateaus and 11 dormant volcanoes, and Mount Cameroon, a currently active volcano (Chauvel et al., 2005). Only three volcanic peaks in the CVL have elevation above 3000 m (Pico Basilé, Mt. Cameroon, and Mt. Oku) (Table 1). The geological structure of the CVL is a combination of tectonic and volcanic origins with unequal ages ranging from the middle to late Tertiary (Jesus et al., 2005). The oldest mountains are in the north, decreasing with the age of volcanic activity in the southern area (Missoup et al., 2016) and then getting older at the oceanic islands in the Gulf of Guinea (Table 1). The marked geographic separation and isolation of the mountains are analogous to islands in the sky or sky islands. Moreover, the sky islands and sky island archipelagos of the Gulf of Guinea and West Central Africa (Figure 1) possess an extraordinary diversity of angiosperms (Figueiredo, 1994), small mammals (Missoup et al., 2016), and amphibians (Zimkus & Gvoždík, 2013). This African region is part of the Guinea biodiversity hotspot (Myers et al., 2000) and is critical for conserving endemic species of plants and animals that inhabit the sky islands (Tropek & Konvicka, 2009).

**TABLE 1:** Elevation and estimated age of six sky islands of the Cameroon Volcanic Line

Sky Island	Elevation (m)	Age (Myr)	Reference
Pico Biao‐Moka	2009	1.3	Yamgouot et al. (2016)
Caldera de Luba	2260	1.3	Yamgouot et al. (2016)
Pico Basilé	3011	1.3	Yamgouot et al. (2016)
Mt. Cameroon	4085	3	Suh et al. (2008)
Bamenda	2621	22–21	Zimkus and Gvoždík (2013)
Mt. Oku	3011	31–22	Suh et al. (2008)

**FIGURE 2:**
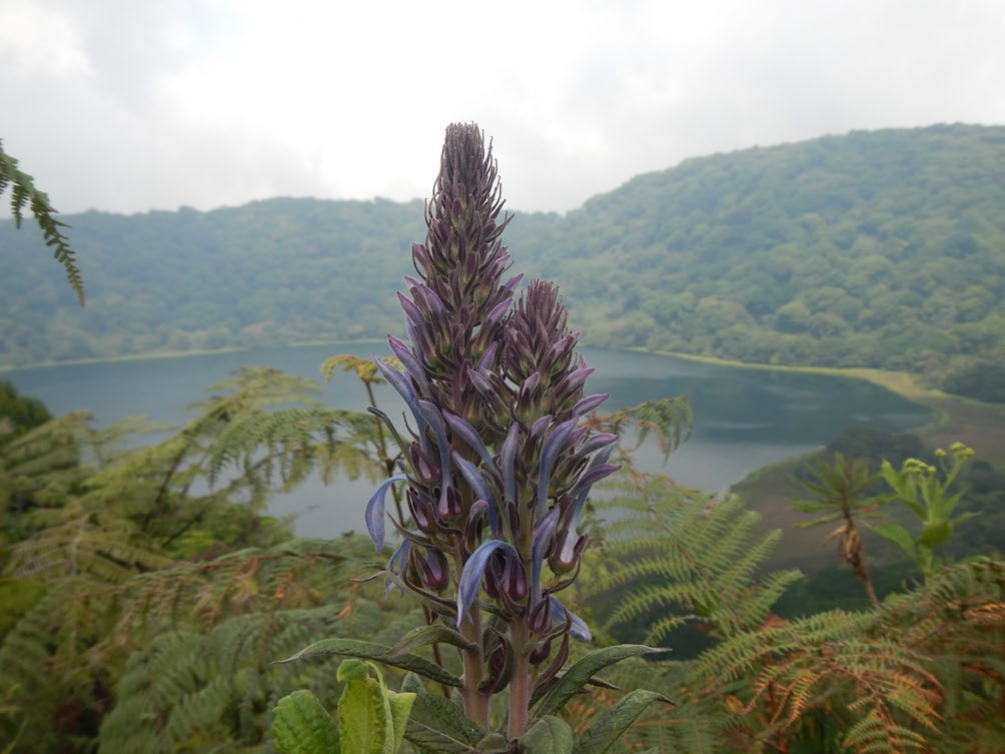
*Lobelia columnaris* at Pico Biao‐Moka, Guinea Equatorial

Giant lobelias may have experienced rapid diversification on East Africa mountains and subsequently dispersed to West Africa (Knox & Li, 2017). *Lobelia columnaris* Hook f. (Campanulaceae: Lobelioideae) is a giant lobelia listed as a vulnerable species in the IUCN Red List of Threatened Species 2015 (Cheek & Thulin, 2015). *L. columnaris* and *L. barnsii* Exell (Mabberley, 1974a) are the two giant lobelia species known from tropical West Central Africa and the Gulf of Guinea. *L. columnaris* (Figure 2) is endemic to Bioko's mountains and highlands (Equatorial Guinea), Nigeria, and Cameroon. This giant lobelia grows in discontinuous populations between 1000 m to 3000 m in different ecological habitats, including submontane grasslands, forest clearings transformed into grasslands overgrown with *Pteridium aquilinum* (L.) Kuhn, and along streams and subalpine meadows.

Little is known about the origin and colonization histories of angiosperms in Bioko and Cameroon. However, a few phylogeographic studies have documented the lineages of plants and animals currently present in Bioko and Cameroon. For example, the Afromontane genus *Lynchis* had several dispersals from Ethiopia and the Western Rift Mountains and recently dispersed to West Africa (Popp et al., 2007). The hypothesis of recurrent connections over time between the West and East African mountains provides a framework to investigate the biogeographic origin among close relatives in both bioregions, like the endangered and endemic Mount Oku rat, *Lamottemys okuensis*, in the CVL (Missoup et al., 2016). However, some taxa have a geographically widespread distribution, from the eastern mainland to the outlying islands of western Africa, like the endangered *Prunus africana* (Dawson & Powell, 2008).

The present study's objective is to infer the phylogenetic relationships of populations of *Lobelia columnaris* using chloroplast genomes and estimate the divergence time to reconstruct its historical colonization in the sky islands of Bioko and Cameroon. Specifically, we aim to answer the following questions: (1) What are the phylogenetic relationships among the Bioko Island and Cameroon populations? (2) Are the older populations found in the older sky islands?

## 2 MATERIALS & METHODS

### 2.1 Study area

Bioko Island lies on the continental shelf and is separated from the Cameroon coast by 32 km of shallow water (60 m). Bioko was separated from mainland Africa 10,000 years ago by the rise of sea level at the end of the last glacial period (Schabetsberger et al., 2004).

A recent study of the geochemistry of volcanic rocks from Bioko dates the main three strato‐volcanoes at <1.3 ± 0.07 Ma; K/Ar (Yamgouot et al., 2016). This dating indicates that the sky islands of Bioko (South and North) are very young with no difference in age. Furthermore, a central depression with a maximum elevation of 500 m separates Pico Basilé (3011 m) in the North and the Pico Biao‐Moka (2009 m) and the Caldera of Luba (2260 m) in the South (Schabetsberger et al., 2004). The vegetation of Bioko is arranged in elevational rings dominated by Guineo‐Congolian tropical species with Afromontane elements appearing at higher elevation (Fa et al., 2000).

Mainland Cameroon comprises several Afromontane sky islands. In the southwest, Mount (Mt.) Cameroon is the highest volcano (4085 m) of West Central Africa. In the northeast, Mount Oku is another high peak with an elevation of 3011 m. Likewise, Bamenda Plateau and Bambutos Mountains represent the orography of the North region. Mountains from the North Central area, such as Mt. Oku and Bamenda‐Banso highlands (2260 m) were uplifted during the Cenozoic (Oligocene–Miocene) (Missoup et al., 2016). Mainland southern volcanoes like Mt. Cameroon are the youngest with origins during the Pliocene–Pleistocene (Zimkus & Gvoždík, 2013). The vegetation is arranged in elevational bands within the montane forest and has been highly disturbed by grazing, fire, and human activities, except on Mt. Cameroon (Ineich et al., 2015).

### 2.2 Study species, DNA extraction, and sequencing

*Lobelia columnaris* Hook f. (Asterales: Campanulaceae: Lobelioideae) is a monocarpic giant rosette plant up to 1 m, with curved purple corollas organized on a terminal or multiterminal panicle. The protandrous flowers are present in the dry season and live approximately 10 days (Bartos et al., 2012). The flowers produce sugared nectar that attracts birds such as the Orange‐tufted sunbird (*Cinnyris bouvieri*) (Janecek et al., 2012; Riegert et al., 2011) and Hymenoptera insects. The flowers are considered bee‐pollinated (Givnish et al., 2009), but birds also can be secondary pollen dispersers. The fruit is a capsule with thousands of winged seeds (Mabberley, 1975) dispersed by wind (Knox & Palmer, 1998; Mabberley, 1975).

Fieldwork was conducted between February and March 2015 with permits from Universidad Nacional de Guinea Ecuatorial, Republic of Equatorial Guinea, and from the Ministry of Scientific Research and Innovation, Republic of Cameroon. Voucher specimens are deposited in the Philadelphia Herbarium (PH). Voucher duplicates are deposited in the National Herbarium of Cameroon (YA) and the Real Jardín Botánico Madrid (MA).

DNA was extracted from silica‐dried leaf samples of 20 individuals of *L. columnaris* using a modified cetyltrimethylammonium bromide (CTAB) protocol (Doyle & Doyle, 1987). Library construction, sequence generation, and bioinformatics processing were done at the Indiana University Center for Genomics and Bioinformatics. Sequence generation was performed using Illumina NextSeq and Illumina MiSeq platforms. Additionally, we downloaded 22 plastome sequences of 22 taxa of Campanulaceae from TreeBASE no. S15797 (Knox, 2014) and GenBank accessions (Knox & Li, 2017).

A total of 20 individual plastid genomes of *L. columnaris* were newly sequenced and assembled (Table 2). Eleven plastomes represent populations from three sky islands in Bioko (Equatorial Guinea), and nine plastid genomes are from two sky islands in Cameroon (Figure 1). Moreover, the phylogenetic tree included two more plastome sequences of *L. columnaris* from South Bioko (Perez Perez 3103, MF061188) and another from Mt. Cameroon (Muasya 2085, MF061187) obtained from GenBank (Knox & Li, 2017). The total dataset included 44 plastomes (22 of *L. columnaris* and 22 from other Campanulaceae taxa).

**TABLE 2:** Locality and collection data of *Lobelia columnaris*

Region	Population (# ind)	GenBank accession number	Sky Island	Elevation (m)
North Bioko	Pz3104_I_2 Pz3104_I_3	MZ428258 MZ428275	Pico Basilé	2947
North Bioko	Pz3105_II_1	MZ428262	Pico Basilé	2836
North Bioko	Pz3106_III_1 Pz3106_III_2	MZ428257 MZ428270	Pico Basilé	2322
South Bioko	Pz3107_IV_2 Pz3107_IV_3	MZ428265 MZ428264	Caldera de Luba	1001
South Bioko	Pz3108_V_1	MZ428273	Pico Biao‐Moka	1582
South Bioko	Pz3109_VI_3	MZ428272	Pico Biao‐Moka	1539
South Bioko	Pz3110_VII_1 Pz3110_VII_3	MZ428259 MZ428261	Pico Biao‐Moka	1157
Cameroon	Pz3111_IX_1	MZ428266	Mt. Oku	2381
Cameroon	Pz3112_X_2 Pz3112_X_3	MZ428256 MZ428268	Mt. Oku	2522
Cameroon	Pz3113_XI_1	MZ428271	Mt. Oku	2197
Cameroon	Pz3114_XII_1	MZ428274	Mt. Oku	1977
Cameroon	Pz3116_VIII_1	MZ428267	Mt. Oku	2407
Cameroon	Pz3117_XIII_1	MZ428269	Bamenda	1798
Cameroon	Pz3118_XIV_2 Pz3118_XIV_3	MZ428260 MZ428263	Bamenda	1723

### 2.3 Assembling and annotation of the chloroplast genomes

The raw paired‐end reads were filtered and de novo assembled using the GetOrganelle toolkit (Jin et al., 2020). The filtered reads were assembled using the SPAdes version 3.9 using *k‐mer* 21, 33, 45, 65, and 85 (Bankevich et al., 2012). To retain pure chloroplast contigs, the final “fastg” files were filtered using the “slim” script of GetOrganelle toolkit. The filtered De Brujin graph was viewed and the final sequence exported using Bandage (Wick et al., 2015). The chloroplast genome was automatically annotated using CpGAVAS (Liu et al., 2012), then adjusted using Geneious version 9.0 (Kearse et al., 2012).

### 2.4 Phylogenetic analyses

The whole chloroplast genome matrix was aligned using MAFFT version 7.1 (Yamada et al., 2016). Only one inverted repeat region was used in the phylogenetic analyses. Two matrixes were prepared, one including all gaps, and another removed gaps using trimAl (Capella‐Gutiérrez et al., 2009) by “‐gt 0.4 ‐st 0.001 ‐cons 40.” Maximum Likelihood (ML) and Bayesian Inference (BI) methods were used to reconstruct phylogenetic trees. No nucleotide positions were excluded from analyses. The ML tree analyses and bootstrap estimation of clade support were conducted with RAxML version 8.2.10 (Stamatakis et al., 2008). These analyses used the GTR substitution model with gamma‐distributed rate heterogeneity among sites and the proportion of invariable sites estimated from the data. Support values for the node and clade were calculated from 1000 bootstrap replicates. Bootstrap support (BS) ≥ 70 is considered as well supported (Hillis & Bull, 1993). The BI analyses were performed using MrBayes version 3.2.6 (Ronquist & Huelsenbeck, 2003), with DNA substitution models selected for each gene partition by the Bayesian information criterion (BIC) using jModeltest version 2.1.10 (Darriba et al., 2012; Guindon & Gascuel, 2003). Markov Chain Monte Carlo (MCMC) analyses were run in MrBayes for 10,000,000 generations, with two simultaneous runs, and each run comprising four incrementally heated chains. The BI analyses were started with a random tree and sampled every 1000 generations. The number of generations for the three datasets was sufficient because the average standard deviation of split frequencies for the datasets was <0.005, and Potential Scale Reduction Factor (PSRF) of Convergence Diagnostic (Gelman & Rubin, 1992) for the datasets was 1.00. The first 25% of the trees were discarded as burn‐in, and the remaining trees were used to generate a majority‐rule consensus tree. Posterior probability values (PP) ≥ 0.95 were considered as well supported (Alfaro et al., 2003; Erixon et al., 2003; Kolaczkowski & Thornton, 2007). Both ML and BI analyses, as well as jModeltest, were performed at the CIPRES Science Gateway (http://www.phylo.org).

### 2.5 Estimation of divergent times and phylogeographic history

Dating analyses were conducted using Markov Chain Monte Carlo (MCMC) methods in BEAST version 2.4 (Bouckaert et al., 2014), which was performed at the CIPRES Science Gateway (http://www.phylo.org). The setting parameters in BEAUti included “BEAST model test” for “Site model,” “Relaxed Clock Log Normal” for “Clock model,” and “Yule Model” for speciation. Meanwhile, we selected two crown nodes for calibrations from published data using the CladeAge package (Matschiner et al., 2016). (1) The *Lobelia thuliniana*—*L. columnaris* clade was 8.0 Mya (sigma 1.0, offset: 0.0), and (2) *Lobelia* laxiflora—*L. columnaris* clade was 21 Mya (sigma 2.0, offset: 0.0) (Chen et al., 2016; Knox, 2014). For reconstructing the phylogeographic history of *L. columnaris*, a plastome phylogeny of the *L. thuliniana–L. columnaris* clade including 22 samples of *L. columnaris* and 5 outgroups was estimated in BEAST2 with “beast‐classic package” of BEAUti. For each dataset, MCMC ran 200,000,000 generations and sampled every 20,000 generations. The first 5000 generations were removed as “Pre Burnin.” Log output of the BEAST analysis was evaluated using Tracer version 1.6. Effective sample sizes (ESS) of all parameters were more than 200, indicating that the estimations were confident. Maximum clade credibility (MCC) tree was generated using TreeAnnotator by setting “Mean heights” for the “Node heights.” The MCC tree was visualized using FigTree version 1.4.2 (http://tree.bio.ed.ac.uk/software/figtree/).

Ancestral area reconstruction was inferred with the Dispersal‐Extinction‐Cladogenesis (DEC) model (Ree & Smith, 2008) implemented in RASP 4.2 (Yu et al., 2020) using the posterior distributions of dated plastome phylogeny that was estimated from the BEAST analysis. The geographic distributions for *Lobelia columnaris* were coded into two areas: (A) Bioko and (B) Cameroon. A third area (C) is represented by *L. giberroa* and *L. stuhlmannii* from East Africa.

## 3 RESULTS

### 3.1 Characteristics of plastomes

**FIGURE 3:**
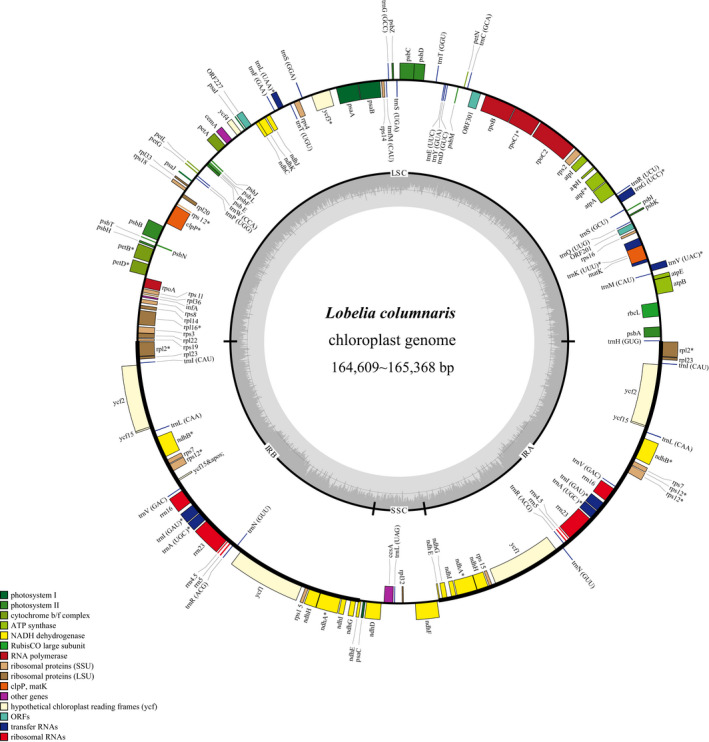
Map of newly sequenced plastome of *Lobelia columnaris*

The plastome of *L*. *columnaris* is circular and quadripartite by having a large single copy (LSC), a small single copy (SSC), and two inverted repeated (IR) regions. The complete plastome varied in size from 164,609 bp to 165,368 bp, with the LSC from 82,421 bp to 82,904 bp, the SSC from 7950 bp to 8025 bp, and the IRs from 37,028 bp to 37,636 bp (Figure 3). The GC content of the whole plastome was 39.2%. The plastome contained 117 unique genes, including 80 recognized and 3 predicted protein‐coding regions (CDS), 30 tRNA, and 4 ribosomal RNA (rRNA) genes. The IR regions had 14 CDS, 7 tRNA, and 4 rRNA genes. The multiple sequence alignment and single plastid genome are deposited in Figshare https://doi.org/10.6084/m9.figshare.14378966. Furthermore, the 20 new annotated plastomes are submitted in GenBank, and their respective GenBank accession numbers are shown in (Table 2).

### 3.2 Phylogenetic relationships

**FIGURE 4:**
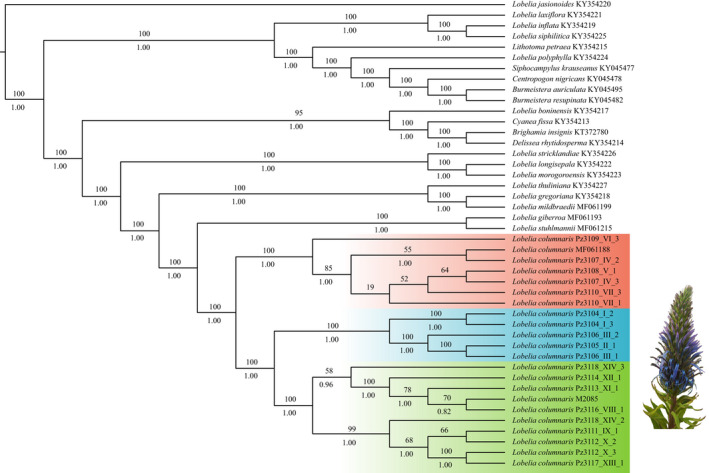
Phylogenomic tree with three highly supported clades of *Lobelia columnaris*. Bootstrap support values (above each branch) and posterior probabilities (below each branch) for the maximum‐likelihood/Bayesian inference tree

The phylogenies from the maximum likelihood (ML) and Bayesian inference (BI) analysis had identical topologies. As expected, all populations of *L. columnaris* sampled from Bioko and Cameroon shared the same ancestor. However, they were arranged in three highly supported clades. Our plastome phylogenetic analysis shows an unexpected result: Bioko has two different clades separated by a short geographic distance. The clade of South Bioko (Pico Biao‐Moka and Caldera de Luba) initially diverged from the other two clades. Moreover, the clades of North Bioko (Pico Basilé) and Cameroon (Mt. Cameroon, Bamenda and Mt. Oku) are more recently diverged (Figure 4).

### 3.3 Events of divergence times

**FIGURE 5:**
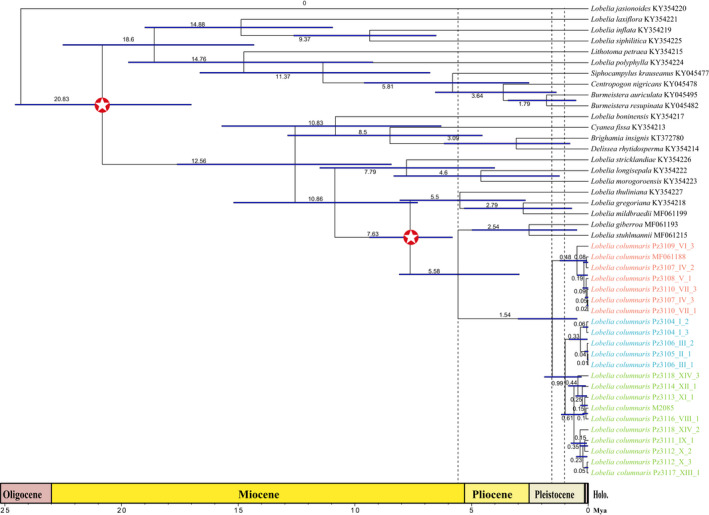
MCC tree with 95% highest probability density (HPD) confidence intervals for phylogenomic relationships and estimation of divergence times obtained in BEAST. The two calibrations are represented by star symbols on the respective internal nodes

Three important divergent events are noticeable on the Bayesian time‐calibrated phylogeny. The first event is estimated as 5.58 Ma with 95% highest probability density (HPD) of 2.95–8.10 Ma, marking the divergence between the ancestor of *L. columnaris* and giant lobelias from East Africa (Figure 5). The second occurred approximately at 1.54 Ma (0.47–3.01 Ma 95% HPD) and marked the divergence of the ancestor of *L. columnaris* in West Central Africa. Finally, the third event is estimated to be 0.99 Ma (0.28–1.88 Ma 95% HPD) displaying the split of North Bioko and mainland Cameroon populations.

### 3.4 Reconstruction of the phylogeographic history

**FIGURE 6:**
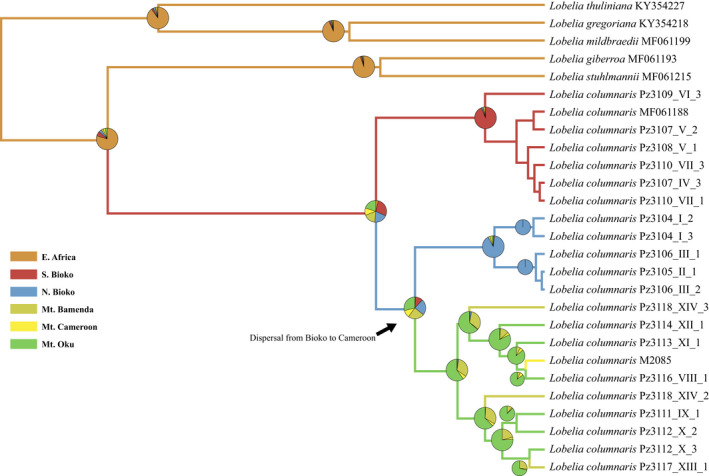
Geographic and phylogenomic relationship of plastomes of *Lobelia columnaris*

**FIGURE 7:**
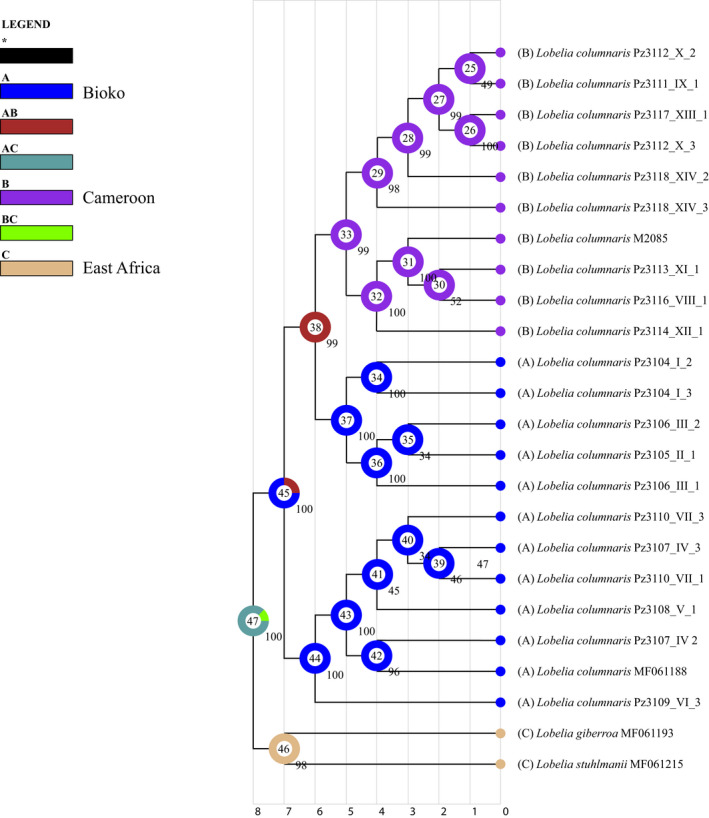
Ancestral area reconstruction of *Lobelia columnaris* using the plastome chronogram obtained in BEAST. A pie chart at each node indicates the possible ancestral distribution inferred from dispersal‐extinction‐cladogenesis (DEC) analysis implemented in RASP

Dispersal events gave origin to the common ancestor of *Lobelia columnaris*. The geographic distribution of plastid genomes in the phylogeny shows that samples were biogeographically reconstructed in the phylogenetic tree. The common ancestor of the populations from Bioko and mainland shows the pie chart with fractions of each different plastome and the geographic regions of the sky islands (Figure 6). Those results show three biogeographic groups. The pie charts of Bioko are restricted to South or North. However, the pie charts with mainland populations show a mix of plastomes genetically similar with a wide geographic connection. A limiting outcome of this analysis was an absence of evidence to infer which peak or sky island was colonized first. Our second biogeographic analysis using the DEC evaluation shows that the ancestral area of *Lobelia columnaris* is South Bioko. Therefore, for this analysis, the populations of Bioko were coded like one region and mainland a second region. The clade of South Bioko had the highest probability (44%). The most probable ancestral area for North Bioko was 37%, and for the clade of Cameroon, the probability was the lowest (33%) (Figure 7).

## 4 DISCUSSION

### 4.1 Phylogenetic relationship of populations

The ancestor of *L. columnaris* probably spread from the Western Rift (East Africa) via Congo's Basin to establish in West Central Africa. Under this scenario, the dispersal event occurred recently (ca. 1.54 Ma [0.47–3.01 Ma 95% HPD]), and the mainland lineage went subsequently extinct (Knox & Li, 2017). Alternatively, one more possible route of dispersal from East to West Africa should include a lineage that colonized the oceanic island of São Tomé and then Bioko. The island of São Tomé is home to the endemic *L. barnsii*. This species looks morphologically similar to *L. columnaris* (personal observation), and both species should be sister species. However, no molecular data that show the position of *Lobelia barnsii* in the phylogenetic tree are available. Once data for that taxon are available, it will help establish the minimum age of colonization of lobelias from East to West Africa (Knox & Li, 2017). Therefore, the alternative dispersal route of the ancestor of *L. columnaris* from East Africa—oceanic island—mainland West Africa is only a hypothesis that needs to be tested.

The genealogical relationships among populations of *L. columnaris* in the present day are better understood with the analyses of plastomes. We hypothesized that the populations on Bioko shared the same common ancestor. Indeed, South and North Bioko share a common ancestral lineage. However, our results clearly show an early colonization event in South Bioko. A subsequent divergence separated the populations of North Bioko and Cameroon.

We found a phylogeographic pattern with three clades distributed on the six sky islands. However, the separation of Bioko's populations into two clades was an unexpected result. This result makes the phylogeography of *L. columnaris*, and probably other organisms present on a relatively small island, complex. A possible explanation about the divergence of North and South clades in Bioko was for the turnover of the plastid genomes by demographic, environmental, or volcanic activity in the sky islands of Bioko. The third clade in mainland Cameroon shares a more recent common ancestor with populations in North Bioko. Moreover, the geographic distance among sky islands in Cameroon does not separate the populations of *L. columnaris* genetically.

### 4.2 Historical colonization and sky island's age

The Hawaiian archipelago is well documented for the correlation between the age of the islands and their colonization and radiation of various plants’ lineages such as the Hawaiian lobeliads (Givnish et al., 2009). In contrast, the West Central Africa sky archipelago does not have a simple chronological age from east to west (Suh et al., 2008), and the whole CVL cannot be compared to the chronological age of the Hawaiian archipelago. Nevertheless, we found that the six sky islands that we studied for *L. columnaris* have a clear progression of age. Their estimated ages run from old (West‐Central Cameroon) to young mountains (Bioko and Mt. Cameroon). Our plastome phylogeographic interpretation indicated that *L. columnaris* colonized the older sky islands in mainland Cameroon after establishing South Bioko's younger sky islands. We recognized with the DEC results using RASP that Bioko was the ancestral area. After this biogeographic analysis, we can infer that Bioko was the cradle for speciation and subsequent dispersal to mainland Cameroon.

Overall, this result contradicts our sky island age hypothesis because Cameroon's sky islands with estimated ages of 3.0, 22.0, and 31.0 Ma are older than Bioko's sky islands (ca. 1.3 Ma) (Table 1). Our results suggest a biogeography history with an inverse correlation with the age of the Afromontane sky islands. There is no simple answer as to why this giant lobelia dispersal history occurred from young peaks or sky islands to old mountains. It is possible that this plant species found a niche at South Bioko that facilitated its survival during critical volcanic and environmental changes in the region. Phylogeography is fascinating because we have much to learn before we can understand the evolutionary biology of organisms, especially those in regions such as West Central Africa, where the climatic and geologic history is very complex.

Perhaps, the South Bioko populations are the sister clade on the ML/BI/BEAST trees because the plastomes provide a skewed view of the biogeographic history of *L. columnaris*, or there is sampling error because we sequenced only one to two individuals per population. Furthermore, we do not have population data from Nigeria, and we did not find more populations in Cameroon, possibly because of local extinction events caused by the intense human transformation of the Afromontane habitat in the sky islands.

It is possible that the incorporation of population‐plastome data from Nigeria will not change the biogeographic patterns found in this study. Because our data show that the ancestral area is in South Bioko, we can predict that populations of *L. columnaris* in Nigeria will be phylogenomically closer to Cameroon with recent colonization history.

Our historical colonization interpretation using plastid genomes and the DEC analysis might be overestimated because the two calibrations used in the estimation of divergence of populations of *L. columnaris* are a little older than the age of the sky islands of our study. However, the colonization history might have underestimated divergence times, but this result will not change the historical dispersal from Bioko to the mainland.

### 4.3 Afromontane forest

Eastern giant lobelias evolved in Afromontane forests at elevation ranges from 1000 to 2500 m. Through frost tolerant adaptations, some taxa colonized the inhospitable Afroalpine elevation from 3000 to 5000 m (Hedberg, 1969). We found that middle elevation is the most likely habitat for *L. columnaris*. Only Pico Basilé’s (North Bioko) population extends its range from middle elevation up to >3000 m. At this elevation, the habitat is composed of shrubs and subalpine meadows.

The paleoclimatic scenarios of the Afromontane vegetation during the Pleistocene in West Central Africa are not well known. However, it is known that climatic fluctuations changed the past vegetation patterns dramatically. With pollen analysis it is possible to reconstruct historical processes (Kadu et al., 2011). Moreover, two broad phylogeographic hypotheses might help understand the complex vegetation structure of Africa's mountains. The first is referred to as the “mountain‐forest bridges” hypothesis (Kebede et al., 2007); this assumes short‐range dispersal between adjacent sky island ranges that facilitated more wide‐ranging forest coverage during warm and humid interglacial periods. The second hypothesis proposes that “long‐distance dispersal” events among isolated mountains during the Pleistocene glacial periods are responsible for the observed phylogeographic patterns of organisms (Mairal et al., 2017). The interglacial and glacial period dynamic allowed species to disperse and be isolated in adjacent mountains (Zimkus & Gvoždík, 2013). Likewise, these environmental fluctuations could cause local and regional extinctions of organisms in general.

Under this scenario, *L. columnaris* probably had changes in population size during the Pleistocene, mainly caused by repeated climatic oscillations and Afromontane fragmentation. Also, environmental fluctuations caused the constriction of populations into forest refugia and changes in geographic distribution. Later, populations expanded by dispersal during warm and humid environmental alterations (Gao et al., 2015). Therefore, the current disjunction of *L. columnaris* in sky islands probably was a Pleistocene product of retraction and isolation of Afromontane forest and continuous post‐Pleistocene dispersal to empty niches. Although not explored in the study, active volcanism and land use have to be considered in a holistic view of the current geographic distribution of this giant lobelia.

### 4.4 Pleistocene refugia

The entire Cameroon line is considered a Pleistocene forest refuge for its distinctive flora. Forest refugia is also supported because of the high genetic diversity detected in different trees (Pineiro et al., 2017), such as the genus *Greenwayodendron* (Migliore et al., 2018), and other flowering plants. For example, *Arabis alpina* (L.) survived the Pleistocene oscillations of temperature and drought in refugia. Once the environmental conditions changed during the interglacial periods, *A. alpina* colonized or recolonized new sky islands in East Central Africa (Assefa et al., 2007).

It is possible that the populations of *L. columnaris* were dynamically isolated and expanded by short‐range dispersal within and between the sky islands of West Central Africa. However, Pico Biao‐Moka in South Bioko might be a Pleistocene forest refuge to Afromontane plant species. Additional landscape changes occurred in the region at the end of the last glacial period (ca. 10,000 years ago) when a rise in sea level isolated Bioko from the African mainland (Jones, 1994). A shallow channel separates Bioko from the Cameroon coast by 32 km (Schabetsberger et al., 2004). We do not have evidence of recent dispersal or gene flow between North Bioko and mainland Cameroon. The central depression with lowland forest (0–500 masl) between North and South Bioko (Schabetsberger et al., 2004) exemplifies another geographic barrier that probably maintains reproductive isolation between the populations of *L. columnaris* in Bioko Island. The central depression consists of lowland forest vegetation.

The expansion, colonization, and recolonization of *L. columnaris* was possible mainly by wind dispersal and possibly by birds. The wind played an essential role in dispersing tiny seeds across sky islands. In Cameroon, high ridges act as a natural forest corridor connecting sky islands and facilitating dispersal and gene flow among contemporaneous populations (Smith et al., 2000).

### 4.5 Ecology and conservation

The type of ecological habitat may have a possible effect on the morphology of *Lobelia columnaris* (Mabberley, 1974b). This observation has to be developed in future studies. Our study observed that populations grow in a mosaic of ecological habitats and elevational gradients. Indeed, we observed phenotypic variation in some traits, for example, in plant height, size and number of inflorescences, leaves, and flower color. *L. columnaris* is smaller in height, inflorescence, and leaf length at higher (approx. 3000 m) and lower elevation (1000 m). At mid elevation, between 2000 and 2600 m, the morphological variation is spectacular with greater height, inflorescence size and number, flower color, and leaf length.

The populations of *L. columnaris* in mainland Cameroon are at high risk of local extirpation because of excessive anthropogenic pressure on montane forest fragments. Only the populations on Mt. Cameroon, which is a National Park, have conservation protection plans. The scenario on Bioko for this giant lobelia and the Afromontane forest is better than the mainland. Bioko has an active conservation procedure for two of the three sky islands (Müller & Pócs, 2007). Moreover, South Bioko is undisturbed because of low human population density and supports the highest numbers of plant and animal species on the island (see Jones, 1994).

### 4.6 Study limits

Several factors limit our results. First, this preliminary study was conducted with few individuals (one to two) for each population, and as such, it should be considered a baseline for future studies. The available sample size for every population is 10 individuals. We hope to conduct future studies with all the individual samples and include samples from Nigeria to better interpret the colonization history of *L. columnaris* in its whole geographic distribution. Second, social instability was the main reason no samples of *L. columnaris* were collected in Nigeria. Future fieldwork in Nigeria to find and collect populations will expand the sampling effort and allow a more robust phylogeographic study. Third, populations from Mt. Cameroon were under‐collected because it was challenging to get the permits to work in the National Park. Finally, this study presents a partial history with the analysis of a nonrecombinant marker. A more robust analysis should include nuclear genomic sequences.

## 5 CONCLUSIONS

Our phylogenetic tree based on plastomes enriched our understanding of the evolution of *Lobelia columnaris* across sky islands of the Cameroon Volcanic Line. According to our phylogenomic calibration, the ancestor of *L. columnaris* arrived in West Central Africa during the Cameroon line's youngest volcanoes’ uplift. The Pleistocene climatic oscillations led to the divergence of the Cameroon and Bioko populations into three clades. Bioko probably was the ancestral area and likely forest refugia during the interglacial periods. Here, we show that the biogeographic history of *L. columnaris* does not follow the progression of ages of the sky islands. In more recent times, Bioko's central depression and the shallow sea channel of 32 km likely function as geographic barriers to further isolate the population groups discovered in our analysis (South Bioko vs. North Bioko and Cameroon). Moreover, grazing, burning, and deforestation are threatening Afromontane forest patches of *L. columnaris* in mainland Cameroon.

## CONFLICT OF INTEREST

None declared.

## AUTHOR CONTRIBUTIONS

**Miguel A. Pérez‐Pérez:** Conceptualization (lead); Funding acquisition (lead); Investigation (lead); Methodology (lead); Project administration (lead); Writing‐original draft (lead); Writing‐review & editing (lead). **Wen‐Bin Yu:** Data curation (lead); Formal analysis (lead); Writing‐review & editing (equal).

## Data Availability

The multiple sequence alignment and single plastid genome were deposited in Figshare https://doi.org/10.6084/m9.figshare.14378966. The 20 new annotated plastomes were submitted to GenBank (for accession numbers see Table 2). Voucher specimens were deposited in the Philadelphia Herbarium (PH). Voucher duplicates were deposited in the National Herbarium of Cameroon (YA) and the Real Jardín Botánico Madrid (MA).
